# A Comparison of Disinfecting Ability of Peracetic Acid, Glutaraldehyde and Autoclaving on Endodontic K-files Tested Against Enterococcus faecalis: An In-Vitro Study

**DOI:** 10.7759/cureus.83168

**Published:** 2025-04-29

**Authors:** Somya Goel, Laresh N Mistry, Ashwin M Jawdekar, Shantanu Deshpande, Minakshi Bhattacharjee

**Affiliations:** 1 Pediatric and Preventive Dentistry, Bharati Vidyapeeth (Deemed to be University) Dental College and Hospital, Navi Mumbai, IND; 2 Microbiology, Bharati Vidyapeeth (Deemed to be University) Dental College and Hospital, Navi Mumbai, IND

**Keywords:** autoclaving, enterococcus faecalis, glutaraldehyde, instrument integrity, k-files, peracetic acid

## Abstract

Aim

This in-vitro study aimed to compare the disinfecting efficacy of peracetic acid (PAA), glutaraldehyde, and autoclaving on endodontic K-files contaminated with *Enterococcus faecalis (E. faecalis), *a resilient pathogen frequently present in persistent root canal infections.

Materials and methods

Twenty-four pre-sterilized nickel-titanium (NiTi) K-files (Mani, Inc., Japan) #10 (25 mm) were infected with a standard strain of *E. faecalis* and randomly divided into three groups (n=8). Group I was treated with 50% PAA (Microgen Hygiene Pvt. Ltd, India) Group II with 2% Glutaraldehyde (3M, India)for 4 hours, and Group III was subjected to autoclaving (Runyes Medical Instrument Co., Ltd., Ningbo, China) at 121°C at 15 lbs of pressure for 15 minutes. Disinfection efficacy was assessed via turbidity testing in peptone water, blood agar plate streaking, and Gram-stain microscopy to detect bacterial presence.

Results

All three disinfecting methods achieved complete microbial eradication. There was no turbidity observed in peptone water, no bacterial colonies on blood agar plates, and no Gram-positive cocci were observed under microscopic examination in any group. As all treatment modalities demonstrated full efficacy, statistical comparison was deemed unnecessary.

Conclusion

PAA, glutaraldehyde, and autoclaving demonstrated equal efficacy in disinfecting *E. faecalis*-contaminated K-files under in-vitro conditions. Given their comparable performance, the choice of sterilization method may be guided by clinical feasibility, cost, and impact on instrument longevity. Further in vivo studies are needed to validate these findings and assess the long-term effects on instrument integrity.

## Introduction

The sterilization and disinfection processes of endodontic instruments are essential for achieving successful outcomes in root canal treatments and for mitigating the risk of cross-contamination. Within the array of instruments utilized in endodontic procedures, K-files serve a crucial function in the mechanical debridement process; however, they exhibit a significant vulnerability to microbial contamination. *Enterococcus faecalis* (*E. faecalis*), a facultative anaerobic Gram-positive bacterium, is often associated with persistent and refractory endodontic infections [1] owing to its exceptional capacity to withstand adverse conditions, infiltrate dentinal tubules, and exhibit resistance to standard disinfectants [2,3]. Considering the resilience of *E. faecalis* within root canal systems, it is essential to implement rigorous sterilization protocols for endodontic files to achieve successful microbial elimination [4].

Conventional approaches to sterilization within the field of dentistry include the utilization of chemical disinfectants as well as various physical sterilization methodologies. Autoclaving is recognized as the optimal method for instrument sterilization, demonstrating efficacy in the eradication of bacterial spores and resilient microbial entities [5]. Exposure to elevated temperatures and steam can induce changes in the physical characteristics of metallic instruments, which may lead to a reduction in their cutting efficiency and mechanical integrity [5]. Conversely, chemical disinfectants, including peracetic acid (PAA) and glutaraldehyde, present an alternative method for achieving sterilization. PAA, recognized for its strong oxidative properties, has become notable due to its extensive antimicrobial effectiveness, swift action, and low levels of toxic residue. Research indicates that this method may serve as a viable substitute for traditional sterilants, especially when organic debris is present [6]. Glutaraldehyde, recognized for its efficacy as a high-level disinfectant, demonstrates significant antimicrobial activity. However, the associated issues of cytotoxicity, potential handling hazards, and environmental implications warrant a comprehensive assessment of its appropriateness for clinical applications [6].

Although various sterilization techniques are accessible, the relative efficacy of these methods concerning endodontic instruments, particularly K-files, has not been sufficiently investigated. Previous studies have predominantly concentrated on assessing the antimicrobial effectiveness of singular disinfectants; nonetheless, there is a scarcity of direct comparative analyses of their efficacy against *E. faecalis* under controlled in-vitro conditions [7-9]. Moreover, although autoclaving is regarded as the standard for sterilization, its relative effectiveness in comparison to chemical disinfectants within real-world clinical environments requires additional research [10].

This study seeks to methodically evaluate the efficacy of PAA, glutaraldehyde, and autoclaving in disinfecting endodontic K-files that have been contaminated with *E. faecalis* under controlled laboratory conditions. This research aims to evaluate the efficacy of various methods in reducing bacterial populations, thereby offering critical insights into the most effective sterilization strategies that achieve complete microbial elimination while maintaining the structural integrity of endodontic instruments.

## Materials and methods

The study was designed to evaluate and compare the disinfecting efficacy of three sterilization methods -PAA, glutaraldehyde, and autoclaving - on endodontic K-files contaminated with *E. faecalis*. The study was an in-vitro study planned in the Department of Pediatric and Preventive Dentistry and carried out in the Department of Microbiology at Bharati Vidyapeeth Dental College and Hospital, Navi Mumbai, from January 2023 to September 2023 (IRB Approval No. 311032022).

Selection and preparation of endodontic files

A total of 24 Niti K-files (Mani, Inc., Japan) of size #10 (25 mm) were selected for the study. This was based on another study conducted by one of the authors [4]. The rationale behind choosing size #10 K-files was based on their position as the first endodontic instruments introduced into the root canal, where they encounter the highest bacterial load and consequently, the maximum infection risk during root canal procedures [11].

Each K-file was pre-sterilized using the standard autoclaving protocol: 121°C at 15 lbs of pressure for 15 minutes [12]. This ensured that the files were free of any contamination prior to infection with the *E. faecalis* strain. This was evaluated on the basis of standardized sterilization strips.

Preparation of *Enterococcus faecalis* culture

The standard *E. faecalis* strain was used to contaminate the files. A microbiological broth culture of *E. faecalis* was prepared according to established protocols. The broth was inoculated and incubated overnight at 37°C for 24 hours to ensure an adequate growth of *E. faecalis* for subsequent infection of the flies [4].

Infection of endodontic files

After the 24-hour incubation period, the *E. faecalis* broth culture was prepared for inoculation onto the K-files. Each pre-sterilized K-file was submerged in a sterile container containing the *E. faecalis* broth for 30 minutes to ensure that the files became thoroughly contaminated. Following this, the files were transferred to sterile petri dishes and incubated at 37°C for 30 minutes. This step was performed to allow for drying and fixation of the microorganisms [4].

Grouping of files and treatment conditions

The files were then randomly divided into three groups to test the efficacy of different disinfecting agents. Each group of eight K-files was subjected to one of the following treatments: Group I (PAA): The #10 K-files were treated with PAA (Microgen Hygiene Pvt. Ltd., India) at a concentration of 50% for 8 minutes, as per the manufacturer’s instructions; Group II (Glutaraldehyde Solution): The #10 K-files were treated with a 2.45% glutaraldehyde solution (MIL Laboratories Pvt. Ltd., India). The files were exposed to the solution for a prolonged period of 4 hours to ensure thorough disinfection; Group III (Autoclaving): The #10 K-files were subjected to autoclaving (Runyes Medical Instrument Co., Ltd., Ningbo, China) at 121°C at 15 lbs of pressure for 15 minutes. This method serves as the gold standard for sterilization in endodontics [13].

In this three-group study, sterilization by autoclaving is the gold standard positive control against which glutaraldehyde, which is considered a standard practice for chairside high-level disinfection and a new material relatively unexplored in endodontic practice, has been compared.

Disinfection efficacy assessment

To assess the efficacy of the disinfecting treatments, three distinct methods were employed to detect the presence of viable microorganisms post-treatment.

Turbidity Method

Each treated file was placed in a test tube containing peptone water. The test tubes were then incubated overnight at 37°C to check for the presence of turbidity, which would indicate bacterial growth. The presence of turbidity would suggest that the disinfecting agent was ineffective in eradicating the microbial load, while a clear solution would indicate successful disinfection [4].

Blood Agar Plate Streaking Method

Following the incubation period, a loopful of the peptone water from each test tube was streaked onto a blood agar plate. The blood agar plates were then incubated at 37°C for 24 hours. The presence of bacterial colonies on the blood agar plate would indicate that *E. faecalis* survived the disinfection treatment, while an absence of colony growth would confirm effective disinfection [4].

Microscopic Examination

For further verification, Gram’s stain was performed on the bacterial sample from the test tube. A smear of the bacterial suspension was prepared on a microscope slide and stained using the standard Gram’s staining technique. The presence of Gram-positive cocci (*E. faecalis*) would be indicative of viable microorganisms remaining on the K-files after disinfection, whereas the absence of these organisms would confirm successful sterilization [4].

Statistical analysis

The results obtained from the turbidity, blood agar streaking, and microscopic examination methods were compared among the three treatment groups. The efficacy of each disinfecting method was determined by the number of positive and negative results for microbial growth, with statistical analysis performed to assess any significant differences in disinfecting efficacy between the treatments. A p-value of less than 0.05 was considered statistically significant. MS Excel was used for data analysis and descriptive statistics.

## Results

Turbidity method

**Figure 1:**
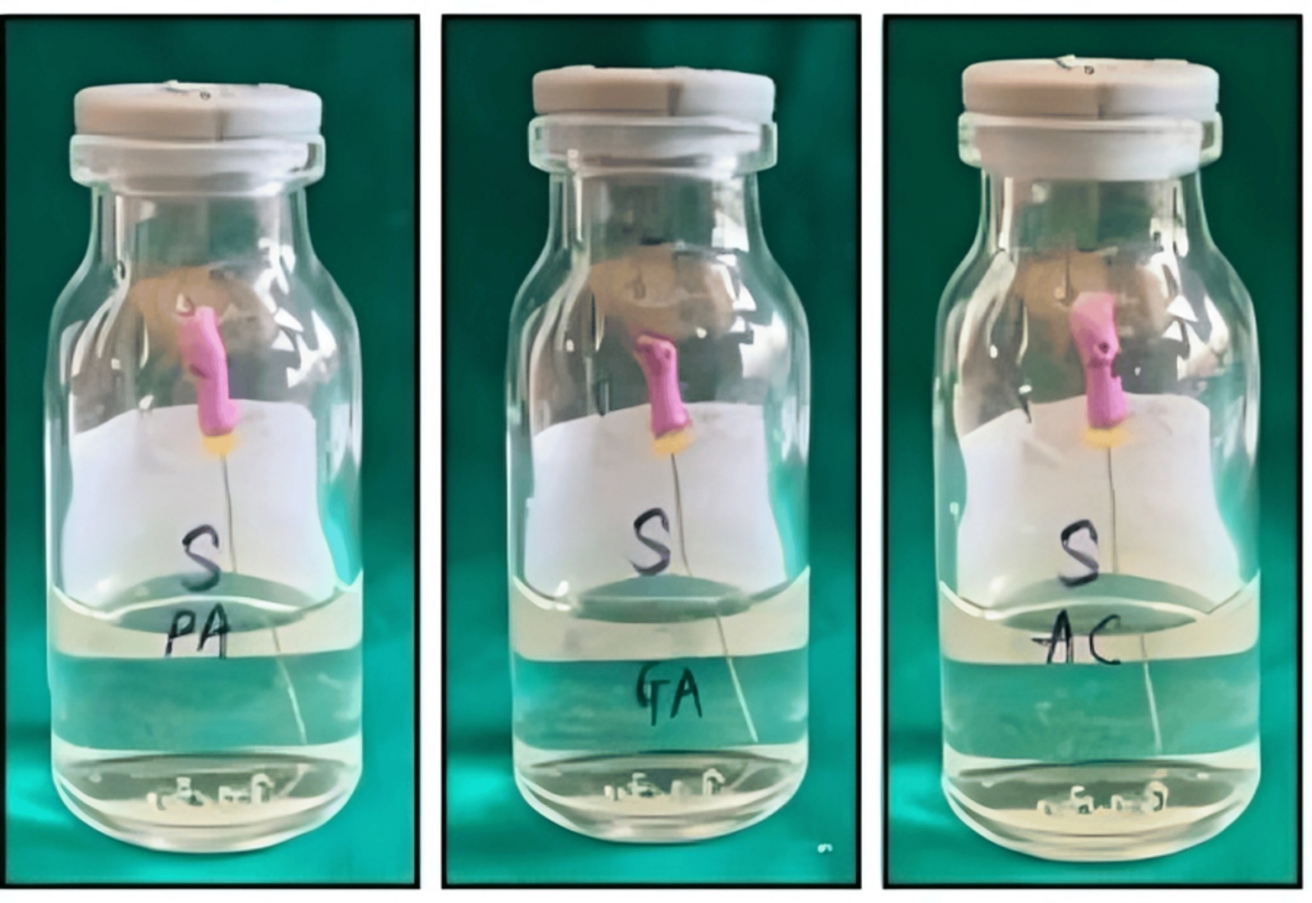
Disinfection of K-files using the turbidity method. The absence of turbidity in the peptone water across all three groups (Group I: PAA, Group II: Glutaraldehyde, Group III: Autoclaving). PAA: peracetic acid

Following the treatment of the K-files with the disinfecting agents, all files were incubated in test tubes containing peptone water for 24 hours at 37°C to assess bacterial growth. The absence of turbidity in the peptone water across all three groups (Group I: PAA, Group II: Glutaraldehyde, Group III: Autoclaving) indicates that no bacterial growth occurred in any of the test tubes (Figure 1). This suggests that the disinfecting agents were effective in eliminating the microbial load on the files, as turbidity is typically indicative of bacterial proliferation. Therefore, all three methods - PAA, glutaraldehyde, and autoclaving demonstrated complete bactericidal action against *E. faecalis*.

Blood agar plate streaking method

**Figure 2:**
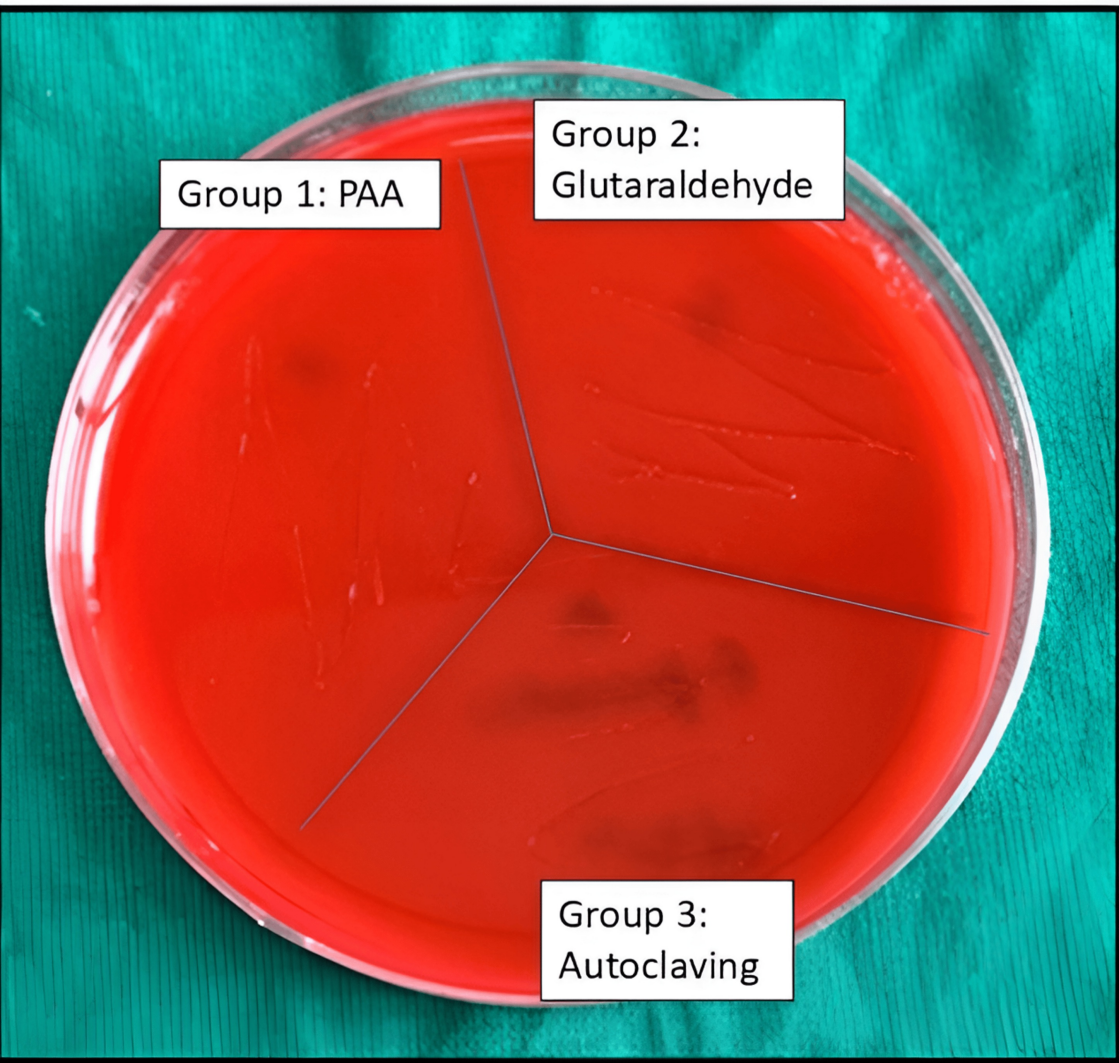
Blood agar plate showing absence of Enterococcus faecalis PAA: peracetic acid

To further confirm the efficacy of the disinfecting treatments, inoculum from the peptone water was streaked onto blood agar plates and incubated for 24 hours at 37°C. No bacterial colonies were observed on any of the blood agar plates from the three treatment groups (Figure 2). This absence of colony growth in all groups strongly supports the findings from the turbidity method, reinforcing the conclusion that the disinfecting agents effectively eradicated *E. faecalis*. The lack of colony formation on the blood agar plates indicates that *E. faecalis* did not survive any of the disinfecting treatments, further validating the efficacy of PAA, glutaraldehyde, and autoclaving in sterilizing the endodontic files.

Microscopic examination

The final verification was performed through microscopic examination, using Gram’s staining to observe the bacterial morphology and confirm the presence or absence of viable microorganisms on the disinfected K-files. The microscopic evaluation revealed no Gram-positive cocci, which are characteristic of *E. faecalis*, in any of the samples. This result confirmed that no viable *E. faecalis* organisms were present on the files after treatment with PAA, glutaraldehyde, or autoclaving. The absence of bacterial cells on the slides further corroborates the findings from the turbidity and blood agar plate methods, establishing that all three disinfecting methods were highly effective in sterilizing the endodontic instruments.

**Figure 3:**
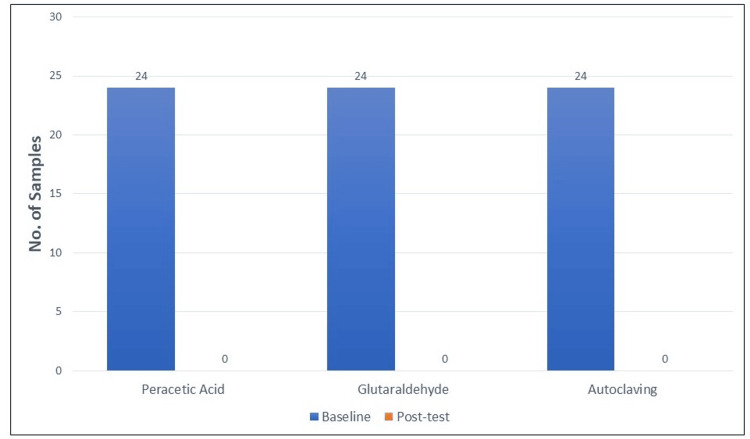
Graph showing the samples infected by Enterococcus faecalis at baseline and post-test.

All three methods demonstrated complete microbial eradication, as evidenced by the absence of turbidity in peptone water, no colony growth on blood agar plates, and no viable *E. faecalis* observed under microscopic examination. All tested disinfection methods exhibited complete eradication of *E. faecalis* as indicated by the lack of bacterial growth across all evaluation techniques (Figure 3). Consequently, inferential statistical tests for comparison do not allow zero values; the p-value-based hypothesis testing is inapplicable, proving all three groups to be equally and 100% effective sterilization methods against *E. faecalis*.

## Discussion

The findings of this investigation indicate that PAA, glutaraldehyde, and autoclaving exhibit comparable efficacy in the disinfection of endodontic K-files that have been contaminated with *E. faecalis*. This observation is consistent with an earlier study that assessed the antimicrobial effectiveness of the sterilization methods in question [6]. *E. faecalis* is frequently identified in instances of failed root canal therapies or scenarios characterized by ongoing inflammation [1,14]. As a result, extensive research has been conducted to evaluate the efficacy of various root canal irrigation solutions against this particular bacterium [15-18]. Considering the resilience of *E. faecalis*, recognized for its capacity to endure extreme conditions, establish biofilms, and withstand standard disinfection methods, it is imperative to attain thorough disinfection of endodontic instruments. This is essential for minimizing the likelihood of enduring infections and enhancing the results of endodontic treatments.

PAA has garnered significant interest as a potent disinfectant, attributed to its extensive antimicrobial spectrum and swift bactericidal efficacy. The available literature provides evidence that PAA exhibited comparable efficacy to sodium hypochlorite and chlorhexidine in the eradication of *E. faecalis*, as assessed through agar diffusion and direct contact assays. The research indicated that a concentration of 2.0% PAA exhibited antibacterial efficacy comparable to that of 5.0% NaOCl, with no viable *E. faecalis* observed following treatment [19]. In a similar investigation, it was demonstrated that PAA exhibited significant efficacy as a root canal disinfectant. However, they raised concerns regarding its potential effects on the cyclic fatigue resistance of nickel-titanium (NiTi) rotary endodontic instruments [20]. Furthermore, PAA demonstrates efficacy in the presence of organic debris, a characteristic that distinguishes it from certain traditional disinfectants. PAA demonstrates a significant capacity to eradicate microbial inocula, encompassing resistant Mycobacterium strains, in a time frame of 10 to 20 minutes, thereby underscoring its efficacy as a high-level disinfectant [6]. The bactericidal efficacy of PAA at a concentration of 50% for 8 minutes was evaluated using the agar diffusion method, yielding comparable results. The mentioned concentration and time were used as per the manufacturer’s instructions. The results of the agar diffusion test conducted in this study indicated that all concentrations of root canal irrigation solutions produced inhibition zones against both bacterial strains examined [21].

Glutaraldehyde is extensively utilized for the disinfection of instruments, attributable to its potent antimicrobial characteristics [22]. Nonetheless, there have been raised concerns pertaining to the cytotoxic properties of glutaraldehyde and the potential health risks it poses to dental professionals [23]. Research conducted by several researchers has identified negative outcomes, including skin irritation, respiratory complications, and environmental implications, associated with prolonged exposure to glutaraldehyde-based disinfectants [4]. Our research indicated a potent disinfection by using 2.45% glutaraldehyde for 4 hours as per the manufacturer’s instructions. Furthermore, recent in-vitro studies indicate that glutaraldehyde exhibits a relatively lower efficacy in sterilization, as persistent microbial growth was noted even after 12 hours of immersion in a 2.4% solution. This observation highlights the limitations of this when applied to the sterilization of critical instruments [24]. Notwithstanding these constraints, its extensive efficacy is unequivocally recognized.

Autoclaving represents the optimal method for sterilization, as it effectively eradicates bacterial spores and resilient microbial entities via the application of high-temperature steam. It is recognized as the most reliable method for ensuring complete microbial elimination [19]. Nonetheless, the research also highlighted apprehensions regarding the possible implications for the mechanical integrity of endodontic files, specifically noting the diminished cutting efficiency of heat-treated NiTi rotary instruments after undergoing multiple autoclaving cycles. Recent findings suggest that autoclaving may lead to a decrease in cyclic fatigue resistance and torsional toughness, which can be attributed to microstructural alterations, surface oxidation, and possible changes in the phase transformation behavior of the alloy [25].

In a research investigation assessing sterilization methodologies, it was demonstrated that autoclaving successfully inactivated *E. faecalis*, including its biofilm-associated state. Nevertheless, the study underscored the potential hazards associated with surface modifications and microfractures in endodontic instruments that experience repeated thermal exposure [26]. Based on the results of this investigation and the comprehensive body of existing research, all three methodologies successfully eradicate *E. faecalis* from K-files. This indicates that dental practitioners might choose a sterilization technique influenced by factors such as financial considerations, user-friendliness, and the durability of instruments, rather than solely on effectiveness.

The primary advantage of this research lies in the implementation of three unique and standardized disinfection techniques, facilitating a comprehensive evaluation of their effectiveness. All methods underwent rigorous testing in a controlled environment, guaranteeing that variables such as bacterial exposure, incubation duration, and assessment criteria were uniform across all experimental groups. The application of methodological rigor significantly improves the reliability of the findings, thereby confirming that the observed differences, or the absence of such, can be directly linked to the disinfection technique employed, rather than being influenced by external confounding variables. Additionally, the research employed *E. faecalis* as the experimental organism, which is well-documented as one of the most formidable endodontic pathogens owing to its capacity to endure in infected root canals even in the face of standard therapeutic interventions.

Notwithstanding its strengths, this study is subject to several limitations. Initially, the lack of bacterial proliferation post-disinfection indicates a successful sterilization process; however, this does not unequivocally confirm the total eradication of all possible infectious agents. The detection methods employed in this investigation were specifically formulated to identify viable *E. faecalis* cells; however, they may not adequately consider the presence of bacterial remnants, biofilms, or residual endotoxins, which could potentially play a role in post-treatment complications. It is observed that *E. faecalis* biofilms demonstrate heightened resistance to disinfectants, a factor that may not have been thoroughly examined in this study [19].

Furthermore, the research did not assess the prolonged implications of recurrent exposure to these disinfection techniques on the structural integrity of K-files. It is proposed that autoclaving could induce structural changes that impact the performance of files over time; however, there is a notable absence of comparable research examining the effects of PAA and glutaraldehyde exposure on the longevity of instruments [20]. Future investigations to examine the effects of multiple sterilization cycles on the longevity of instruments and their cutting performance should be performed.

Another limitation is that the investigation was performed in an in-vitro setting, which may not completely emulate the clinical environment. In-vitro studies yield significant insights regarding the efficacy of disinfection within controlled environments; however, they fail to consider variables such as the existence of organic debris, dentinal tubules, or the responses of the host immune system. Subsequent investigations ought to corroborate these results within clinical contexts.

Clinical significance

The results of this study present considerable clinical implications for the field of endodontics. Given that PAA, glutaraldehyde, and autoclaving exhibit similar effectiveness in the disinfection of K-files, it is reasonable for practitioners to select a sterilization method that aligns with logistical and economic factors. Although autoclaving is widely regarded as the optimal sterilization technique in numerous clinical environments, PAA and glutaraldehyde present viable alternatives, especially in scenarios where autoclaving is impractical. For practices aiming to optimize resource allocation, glutaraldehyde continues to be a feasible option, notwithstanding its associated toxicity issues. In contrast, PAA exhibits a swift bactericidal effect while producing minimal residue, thereby presenting itself as a compelling choice.

## Conclusions

This investigation presents substantial evidence indicating that PAA, glutaraldehyde, and autoclaving demonstrate equivalent efficacy in the disinfection of endodontic K-files contaminated with *E. faecalis*. The results underscore the critical role of stringent sterilization protocols in endodontics and emphasize the necessity for additional investigation into the durability of instruments after multiple disinfection cycles. In light of the constraints inherent to in-vitro research, this investigation highlights the critical significance of choosing a suitable sterilization method, taking into account clinical requirements, the preservation of instruments, and the preferences of the practitioner.
